# Human Pulmonary Artery Endothelial Cells Increased Glycolysis and Decreased Nitric Oxide Synthase O-GlcNAcylation in Pulmonary Arterial Hypertension

**DOI:** 10.3390/ijtm4010007

**Published:** 2024-02-02

**Authors:** Sarah E. Basehore, Alisa Morss Clyne

**Affiliations:** 1 School of Biomedical Engineering, Science, and Health Systems, Drexel University, Philadelphia, PA 19104, USA; 2 Fischell Department of Bioengineering, University of Maryland, College Park, MD 20742, USA

**Keywords:** pulmonary hypertension, endothelial cell, nitric oxide, sex differences, glycosylation, glycolysis, metabolism

## Abstract

Pulmonary arterial hypertension (PAH) is a fatal disease that primarily affects women. In PAH, endothelial cells become dysfunctional, reducing production of the vasodilator nitric oxide while increasing proliferation. Other studies suggest altered glucose metabolism in PAH. Our recent study showed that increased endothelial glucose metabolism in disturbed flow increased O-GlcNAcylation of endothelial nitric oxide synthase (eNOS), the enzyme that makes nitric oxide, which then reduced nitric oxide production. We therefore hypothesized that elevated endothelial glycolytic activity in PAH endothelial cells would reduce nitric oxide production by increasing eNOS O-GlcNAcylation. We cultured human pulmonary artery endothelial cells (HPAECs) from failed lung transplant (“non-PAH”) and idiopathic PAH patients (“PAH”) and quantified glycolytic activity, nitric oxide production, and eNOS O-GlcNAcylation in each cell type. Our data show that PAH HPAECs had higher glucose uptake and glycolytic metabolites, as well as decreased nitric oxide production, compared to non-PAH HPAECs. However, PAH HPAECs had lower eNOS O-GlcNAcylation and UDP-GlcNAc, the substrate for O-GlcNAcylation. Interestingly, both glucose uptake and eNOS O-GlcNAcylation were higher in female as compared to male HPAECs. These data suggest that although endothelial glycolytic metabolism is altered in PAH, eNOS O-GlcNAcylation is not connected to decreased nitric oxide. In addition, differences in glucose metabolism and protein O-GlcNAcylation in HPAECs from male and female donors could relate to PAH sexual dimorphism.

## 1. Introduction

Pulmonary arterial hypertension (PAH) is a chronic, progressive disease leading to right heart failure and death [1]. PAH has an estimated prevalence of 1% globally [2] and predominantly affects female patients [3]. Despite treatment advances, PAH has no cure, and the three year survival rate is only 54.5% [1]. PAH pathology includes imbalanced vasoconstriction and vasodilation, as well as imbalanced cell proliferation and apoptosis [4,5]. Dysfunctional endothelial cells contribute to PAH pathology by producing less of the vasodilator nitric oxide and by losing their contact-inhibited monolayer phenotype and instead proliferating into the vascular lumen [6-11]. Endothelial cells in the resulting plexiform lesions show an increased expression of proliferative markers such as Ki-67, angiogenic molecules such as vascular endothelial growth factor (VEGF), and survival-related molecules such as hypoxia inducible factor (HIF)-1α [12-15].

Endothelial nitric oxide is of particular interest in PAH and has been mechanistically linked to vascular tone and remodeling in PAH pathogenesis. PAH patients have decreased pulmonary nitric oxide as compared to healthy controls [16]. Endothelial cells in PAH patients show decreased nitric oxide [6] as compared to healthy controls. Mice deficient in endothelial nitric oxide synthase (eNOS), the enzyme that produces nitric oxide, also show increased pulmonary arterial pressure which can be abrogated by nitric oxide [17,18].

While multiple pathogenic pathways have been linked to PAH [19], recent studies suggest that PAH pathology includes metabolic dysregulation [20-22]. Endothelial cells in PAH patients show increased glucose metabolism [10] as compared to healthy controls. Pulmonary cells may shift from oxidative phosphorylation to glycolysis [23], similar to cellular metabolic phenotypes observed in cancer [24-26]. PAH patients also showed increased pulmonary ^18^F-labeled deoxyglucose uptake on PET/CT when compared to healthy controls, indicating increased lung glucose uptake [27].

When glycolytic flux increases, metabolic flux down glycolytic side branch pathways such as the hexosamine biosynthetic pathway (HBP) may also increase. First, the rate limiting enzyme glutamine:fructose-6-phosphate amidotransferase converts the glycolytic intermediate fructose-6-phosphate (F6P) to glucosamine-6-phosphate (G6P). G6P is then used to form UDP-GlcNAc, which provides the substrate for the protein O-GlcNAcylation of around 4000 cytoplasmic and nuclear proteins. GlcNAc is added to or removed from protein serine and threonine residues by O-GlcNAc transferase (OGT) and O-GlcNAcase (OGA) [28].

Several groups have studied the adverse effects of chronically elevated *O*-GlcNAc in cardiovascular disease, with a focus on hyperglycemia and diabetes [29-34]. High glucose increases protein GlcNAcylation in the human aorta, coronary artery endothelial cells, umbilical vein endothelial cells, cardiomyocytes, and skeletal muscle [31,35-38]. In PAH, pulmonary artery smooth muscle cells showed increased HBP flux and enhanced OGT [20]. Elevated endothelial protein GlcNAcylation is linked to decreased angiogenesis and increased inflammation [39-41]. O-GlcNAcylation also affects nitric oxide production by decreasing eNOS activation. In hyperglycemia, eNOS glycosylation through the HBP doubled while eNOS activation via serine 1177 phosphorylation decreased proportionally [42]. We also recently showed that endothelial cells in oscillating disturbed flow have increased eNOS O-GlcNAcylation, which leads to decreased nitric oxide production [43]. Thus, eNOS O-GlcNAcylation may contribute to cardiovascular disease, including possibly PAH.

The impact of increased glucose metabolism on endothelial O-GlcNAcylation and nitric oxide production in PAH has not yet been investigated. We hypothesized that elevated endothelial glycolytic activity would reduce nitric oxide production by increasing eNOS O-GlcNAcylation. We therefore cultured human pulmonary artery endothelial cells (HPAECs) from failed lung transplant (“non-PAH”) and idiopathic PAH patients (“PAH”). We quantified glycolytic activity, nitric oxide production, and eNOS O-GlcNAcylation in each cell type. This study highlights important differences in glycolytic activity and eNOS O-GlcNAcylation in PAH endothelial cells from male and female patients, which could contribute to PAH pathology.

## 2. Materials and Methods

### 2.1. Cell Culture

Human pulmonary artery endothelial cells (HPAECs) from non-PAH (donor lungs that were not able to be transplanted) and idiopathic PAH (IPAH; obtained at the time of lung transplant) patient lungs were obtained from the Pulmonary Hypertension Breakthrough Initiative (PHBI) Research Network. We received *n* = 6 donors (3 female, 3 male) for both non-PAH (H1–H6) and PAH (P1–P6) HPAECs (Table 1). Cells (passages 6–10) were cultured in Microvascular Endothelial Growth Medium-2 (EGM-2MV; Lonza, Basel, Switzerland) supplemented with 10% fetal bovine serum (FBS; HyClone, Logan, UT, USA), 1% penicillin–streptomycin, and 1% L-glutamine (ThermoFisher Scientific, Waltham, MA, USA) on gelatin-coated (ESGRO 0.1% gelatin solution, Sigma-Aldrich, St. Louis, MO, USA; #SF008) dishes. All cells were maintained in a humidified environment at 37 °C and 5% CO_2_, with a medium change every two days.

### 2.2. Metabolic Analysis

A YSI 2950 Bioanalyzer (Xylem, Washington, DC, USA) was used to measure changes in glucose and lactate in the cell culture medium. Non-PAH and PAH HPAECs were seeded at confluence and maintained in static culture for 48 h, after which a media sample from each sample was collected. The media sample was centrifuged at 1000 RPM for 5 min to remove any cellular debris. The supernatant was then collected and analyzed on the YSI Bioanalyzer. Glucose uptake and lactate secretion were calculated as the difference between the initial and final glucose and lactate concentrations. Samples were run in triplicate for each sample (three technical replicates), and the average of the technical replicates for each donor sample is shown in the analysis.

HPAEC metabolites were measured via ^13^C_6_-glucose liquid chromatography mass spectrometry (LC-MS). HPAECs were grown to confluency, after which 5 mM ^13^C_6_-glucose (Cambridge Isotope Laboratories, Tewksbury, MA, USA; CLM-1396) in glucose-, glutamine-, and pyruvate-free DMEM (ThermoFisher Scientific, Waltham, MA, USA; A14430-01) supplemented with 10% dialyzed FBS, 1% penicillin–streptomycin, and 1% L-glutamine was added to cells for 2 min. The ^13^C_6_-glucose media was removed, and ice-cold 80:20 methanol/water was added to cells for 15 min at −80 °C. Cells were scraped off the surface, and cell lysates were pipetted into Eppendorf tubes. Samples were centrifuged at 16,000× *g* for 10 min at 4 °C to pellet cell debris. The supernatant was then desiccated under nitrogen and re-dissolved in LC-MS-grade water. Metabolites were analyzed via reverse-phase ion-pairing chromatography coupled to an Exactive Orbitrap mass spectrometer (ThermoFisher Scientific, Waltham, MA, USA) following an established protocol [44].

### 2.3. Protein Analysis

Protein levels were determined via Western blot. HPAECs were lysed with RIPA buffer (ThermoFisher Scientific, Waltham, MA, USA; #89900) and then needle lysed by passing the samples through a 25 gage needle three times. Samples were then centrifuged for 10 min at 10,000× *g* and 4 °C to remove insoluble material. Cell lysates were normalized for protein content by BCA Assay (ThermoFisher Scientific, Waltham, MA, USA), separated by SDS-PAGE on a 4–12% Bis-Tris gel (ThermoFisher), and transferred to a nitrocellulose membrane (ThermoFisher Scientific, Waltham, MA, USA). After blocking in 5% bovine serum albumin (BSA, Sigma-Aldrich, St. Louis, MO, USA), membranes were incubated with primary anti-bodies for GlcNAc CDT110.6 (9875S), OGT (24083), and OGA (SAB4200267 Sigma-Aldrich, St. Louis, MO, USA) at 1:1000 dilution overnight at 4 °C followed by the appropriate secondary horseradish peroxidase-conjugated antibody (1:2000, Promega, Madison, WI, USA) for 1 h at room temperature. β-actin (SC47778-C4, Santa Cruz, Dallas, TX, USA) was the loading control. Protein bands were detected using an enhanced chemiluminescence kit (Western Lightning, PerkinElmer, Waltham, MA, USA) and visualized with a Fluorchem digital imager (Alpha Innotech, San Leandro, CA, USA). Band intensity was quantified using AlphaEase FC software V4.0.

### 2.4. Nitric Oxide

HPAEC nitric oxide was measured via a Griess Assay (Thermofisher Scientific, Waltham, MA, USA; G7921). Cells were incubated for 24 h prior to media collection. Nitrate reductase (20U, NECi Superior Enzymes, Lake Linden, MI, USA; AtNA-R-3U) was added to the collected HPAEC media for 30 min at 37 °C. Media samples were combined with freshly prepared Griess reagent in a 96-well plate as per the manufacturer’s instructions. The plate was then incubated at room temperature for 30 min. Sample absorbance was measured at 548 nm using a Biotek Synergy H1 Microplate Reader. Nitrite was quantified using a nitrite standard curve.

### 2.5. Statistical Analysis

Statistical analysis was performed with GraphPad Prism. Each experiment was repeated at least two times. Comparisons between two groups were analyzed by Student’s *t*-test, and comparisons among multiple groups were analyzed by analysis of variance (ANOVA) with a Bonferroni post hoc test. Statistical significance is indicated by # *p* < 0.05, * *p* < 0.01, ** *p* < 0.001, *** *p* < 0.0001, or **** *p* < 0.00001.

## 3. Results

### 3.1. PAH HPAECs Had Higher Glucose Uptake with Reduced Lactate Secretion

**Figure 1.**
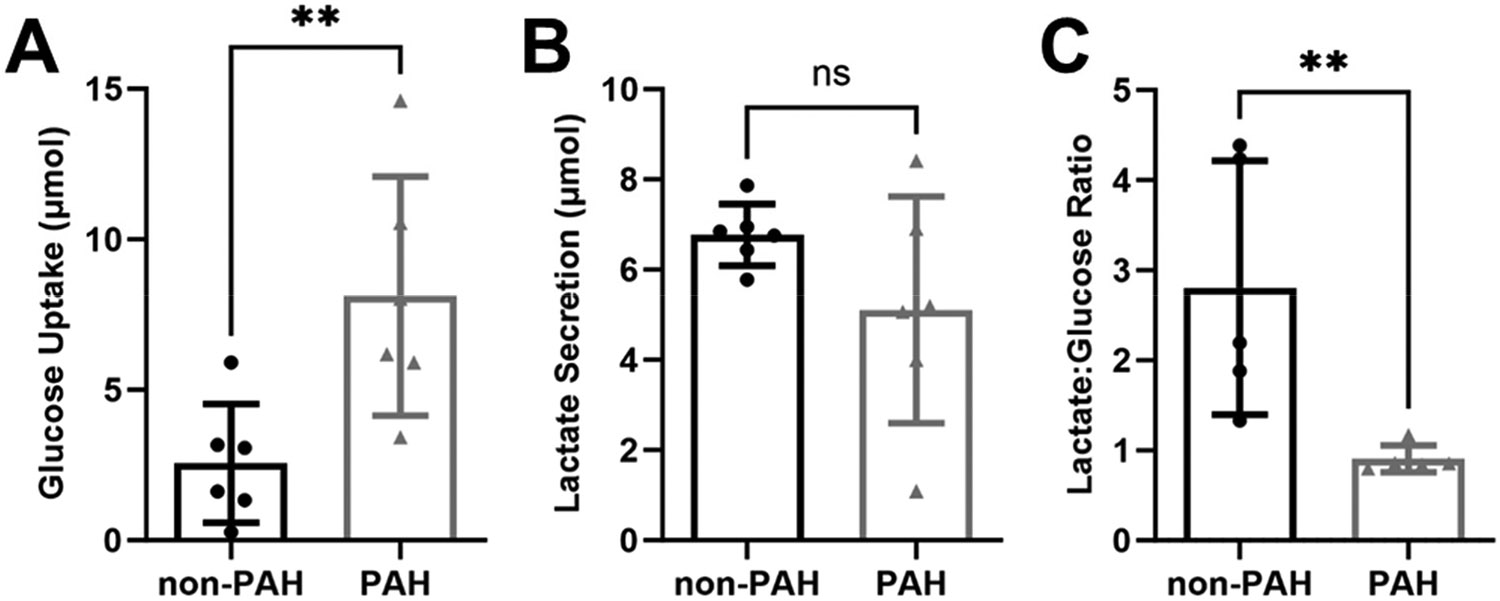
PAH HPAECs consumed more glucose and produced less lactate compared to non-PAH HPAECs. (**A**) Glucose uptake and (**B**) lactate secretion in non-PAH (black circles) and PAH HPAECs (gray triangles) measured via YSI Bioanalyzer. *n* = 6 human donors per condition. (**C**) Lactate/glucose ratio for non-PAH and PAH HPAECs. *n* = 5 human donors per condition, with one outlier for each condition omitted. ns: not significant; ** *p* < 0.001.

We previously observed increased endothelial glycolytic activity in cells exposed to disturbed flow, which led to decreased nitric oxide via increased eNOS O-GlcNAcylation [43]. We therefore examined glucose metabolism in HPAECs from non-PAH and PAH patients to determine if endothelial cells from people with PAH also had increased glycolytic activity. PAH HPAECs showed more than three times higher glucose uptake (*p* < 0.001) compared to non-PAH HPAECs (Figure 1A), as measured via a YSI Bioanalyzer. PAH HPAEC lactate secretion also trended lower, although this change was not statistically significant (Figure 1B). The lactate/glucose ratio was significantly lower in PAH HPAECs (*p* < 0.01; Figure 1C) when two patients were excluded as outliers, as determined by Grubb’s test (*p* < 0.05). These data show that PAH HPAECs consume more glucose yet produce less lactate compared to non-PAH HPAECs.

**Figure 2.**
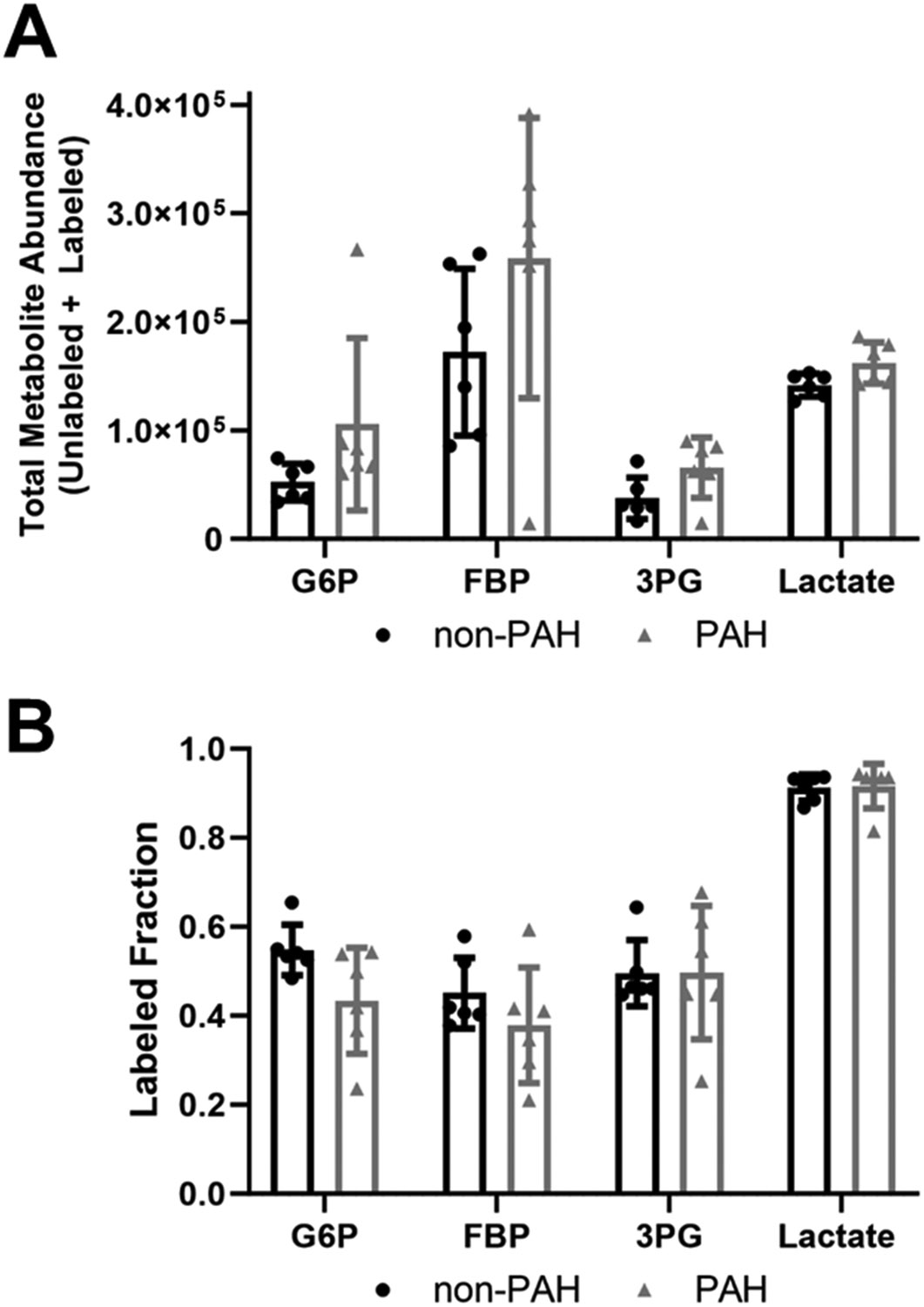
Total glycolytic metabolite abundances were higher in PAH HPAECs than non-PAH HPAECs but the labeled fraction did not change. (**A**) Total metabolite abundances (labeled and unlabeled) and (**B**) labeled fraction (labeled/total) for glucose-6-phosphate (G6P), fructose-1-6-bisphosphate (FBP), 3-phosphoglycerate (3PG), and lactate measured by ^13^C_6_ glucose mass spectrometry in non-PAH and PAH HPAECs. *n* = 6 human donors per condition.

Since overall glycolytic flux increased in PAH HPAECs, we then used ^13^C_6_ glucose mass spectrometry to quantify total glycolytic metabolite pools (unlabeled and labeled) as well as the labeled fraction (relative flux). All glycolytic metabolite total pools increased in PAH HPAECs as compared to non-PAH HPAECs (Figure 2A), although these changes were not statistically significant due to high variability. The glycolytic metabolite labeled fraction, which indicates the percent of the glycolytic metabolite labeled with the heavy isotope, did not differ between non-PAH and PAH HPAECs (Figure 2B). These data suggest that relative glucose flux in glycolysis did not change in PAH HPAECs, but rather that increased glucose uptake contributed to higher glycolytic intermediates in PAH HPAECs.

### 3.2. PAH HPAECs Had Lower Nitric Oxide Production Yet Lower eNOS O-GlcNAcylation

**Figure 3.**
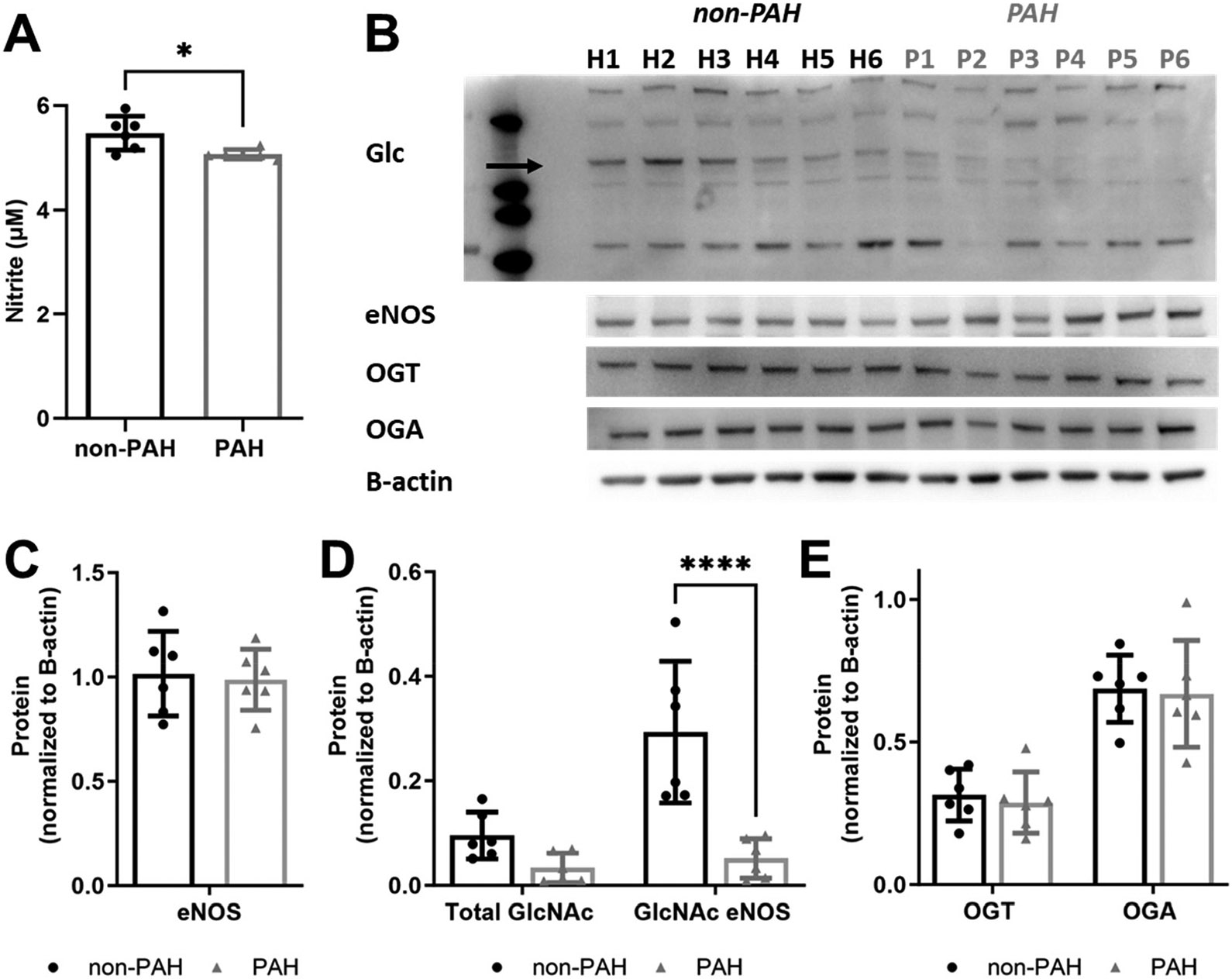
PAH HPAECs produced less nitric oxide yet had less GlcNAcylated eNOS compared to non-PAH HPAECs. (**A**) Nitrite concentration, measured via Griess assay, for non-PAH vs. PAH HPAECs over 24 h. (**B**) Western blot for all O-GlcNAcylated proteins (eNOS is indicated by the arrow), eNOS, OGT, and OGA. Arrow indicates eNOS. Quantification of (**C**) eNOS, (**D**) total GlcNAcylated protein, and GlcNAcylated eNOS and (**E**) OGT and OGA. *n* = 6 human donors per condition. * *p* < 0.01, **** *p* < 0.00001.

Since patients with PAH have reduced nitric oxide levels [6,7,16], we measured endothelial nitric oxide production in non-PAH and PAH HPAECs. Nitrite decreased by about 7.5% in PAH HPAECs (*p* < 0.01) as compared to non-PAH HPAECs (Figure 3A). However, PAH HPAECs showed ~65% lower total O-GlcNAcylated proteins and more than 80% lower eNOS O-GlcNAcylation (*p* < 0.0001; Figure 3B,C). This occurred without a change in OGT or OGA protein (Figure 3D).

**Figure 4.**
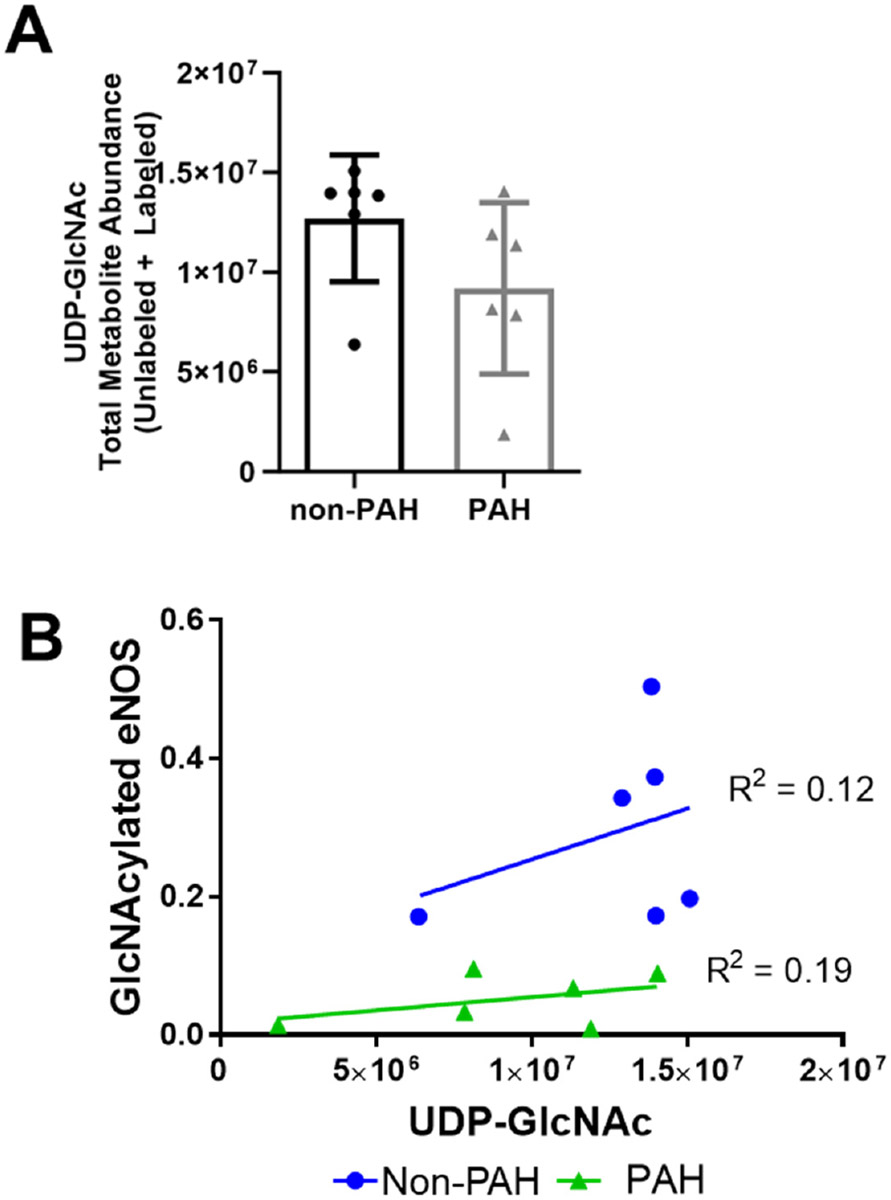
UDP-GlcNAc total metabolite abundances were lower in PAH HPAECs compared to non-PAH HPAECs. (**A**) UDP-GlcNAc total abundance measured by mass spectrometry in non-PAH and PAH HPAECs. *n* = 6 human donors per condition. (**B**) Correlation analysis of GlcNAcylated eNOS versus UDP-GlcNAc levels for non-PAH and PAH HPAECs.

Since protein O-GlcNAcylation also depends on UDP-GlcNAc substrate availability, we next examined UDP-GlcNAc quantity via mass spectrometry. The UDP-GlcNAc total metabolite abundance was approximately 30% lower in PAH HPAECs (Figure 4A) as compared to non-PAH HPAECs, although this change was not statistically significant due to high variability. Indeed, when we plotted GlycNAcylated eNOS vs. UDP-GlcNAc for each donor, UDP-GlcNAc did not correlate with O-GlcNAcylated eNOS (Figure 4B) for non-PAH (R^2^ = 0.12) or PAH HPAECs (R^2^ = 0.19). These results suggest that the UDP-GlcNAc substrate may decrease in PAH patients, but there are other factors contributing to the overall lower eNOS O-GlcNAcylation.

### 3.3. HPAEC High Donor Variability Could Be Linked to Sex Differences

**Figure 5.**
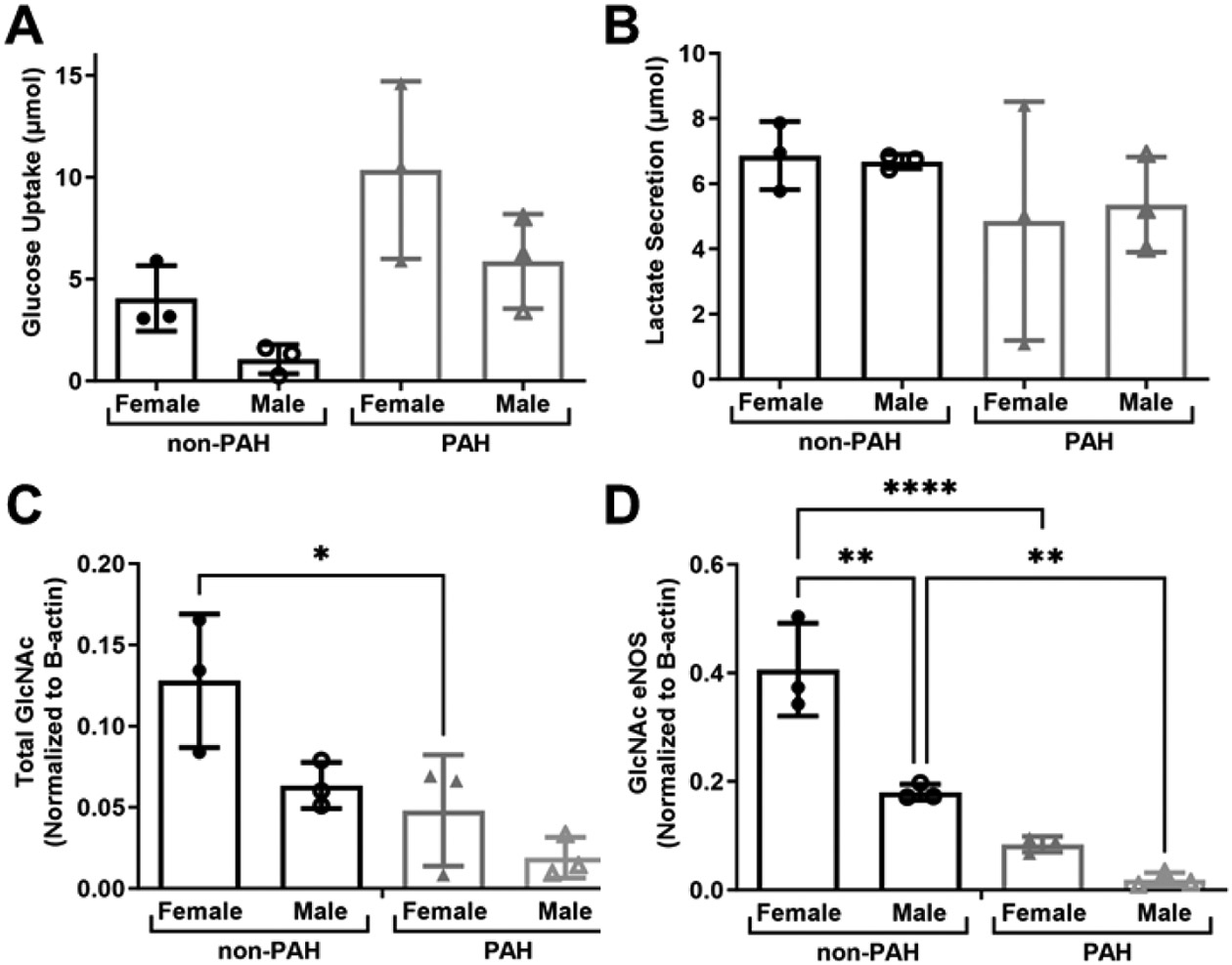
HPAECs from female donors had higher protein and eNOS O-GlcNAcylation. (**A**) Glucose uptake and (**B**) lactate secretion in HPAECs from female and male non-PAH and PAH donors measured via YSI Bioanalyzer. (**C**) Quantification of total GlcNAcylated protein and (D) GlcNAcylated eNOS from female and male non-PAH and PAH donors. *n* = 6 human donors per condition. * *p* < 0.01, ** *p* < 0.001, **** *p* < 0.00001.

Since we observed high donor variability in this study, we separated the data by donor sex to determine how sex may affect endothelial glycolytic metabolism and protein O-GlcNAcylation. HPAECs from female donors had higher glucose uptake (Figure 5A). In contrast, lactate secretion was similar between sexes (Figure 5B). Both total protein and eNOS O-GlcNAcylation were higher in female as compared to male donors (Figure 5C,D). Total GlcNAcylated protein was statistically significantly higher in non-PAH female as compared to PAH female donors (*p* < 0.01). GlcNAcylated eNOS was statistically significantly higher for both non-PAH female (*p* < 0.00001) and male (*p* < 0.001) donors as compared to HPAEC donors. Thus, there are likely important sex differences in glucose metabolism and eNOS O-GlcNacylation, which may contribute to sexual dimorphisms in PAH.

## 4. Discussion

PAH is linked to increased glucose metabolism, yet little is known about endothelial glucose metabolism and eNOS O-GlcNAcylation in this disease [20-22]. We hypothesized that elevated endothelial glucose metabolism would increase eNOS O-GlcNAcylation, contributing to reduced endothelial nitric oxide production in PAH. While HPAECs from PAH patients did show elevated glucose uptake and glycolytic activity, as well as decreased nitric oxide production as expected, UDP-GlcNAc and eNOS O-GlcNAcylation decreased in PAH HPAECs. It is therefore likely that different mechanisms drive decreased nitric oxide in PAH. We also found sex differences in glucose uptake and eNOS O-GlcNAcylation, which could relate to different PAH mechanisms in women vs. men.

In our study, PAH HPAECs increased glucose metabolism in vitro, which largely agrees with human studies showing higher pulmonary 18F-labeled deoxyglucose uptake in PAH patients when compared to non-PAH controls, indicating increased lung glucose uptake [27]. Although PAH HPAECs increased glucose uptake, they produced less lactate in our studies in vitro. This is in contrast to elevated lactate in lung tissues of a fetal lamb pulmonary hypertension model [45]. The difference could lie in the fact that this study measured lactate changes in the entire lung, whereas we measured lactate in pulmonary artery endothelial cells specifically. Therefore, the increased lactate in their study may be from lung epithelial or smooth muscle cells rather than endothelial cells. PAH HPAECs may shuttle pyruvate into the mitochondria for oxidative respiration, as we have previously shown can occur in metabolically altered endothelial cells, rather than converting pyruvate to lactate [46,47]. Alternatively, PAH HPAECs may increase glucose flux down glycolytic side branch pathways, such as the pentose phosphate pathway.

Surprisingly, total protein and eNOS O-GlcNAcylation were lower in PAH HPAECs compared to non-PAH HPAECs, despite elevated glucose uptake and glycolytic activity. This is the opposite of what we what we observed in our prior study of endothelial cells in steady laminar and oscillating disturbed flow [43,48]. A previous study found elevated HBP flux, total O-GlcNAcylation, and OGT in pulmonary arterial smooth muscle cells and human IPAH patient lung tissues [20]. Our data suggest that the overall change in human lung lysate O-GlcNAcylation was related to smooth muscle cells and perhaps epithelial cells rather than the endothelium. Additionally, higher eNOS O-GlcNAcylation in non-PAH compared to PAH HPAECs did not relate to OGT or OGA, similar to what we observed in our prior study [43]. Since the amount of UDP-GlcNAc substrate did not correlate with lower O-GlcNAcylation, additional factors must play a role in PAH.

Nitric oxide production was lower in PAH HPAECs despite lower eNOS O-GlcNAcylation. Our data agree with a study in which isolated endothelial cells from a hypoxia-induced pulmonary hypertension rat artery decreased nitric oxide production, measured via DAF-2T fluorescence, as well as reduced eNOS phosphorylation at Ser1177, measured via SDS-PAGE [49]. Thus, a mechanism other than O-GlcNAcylation may reduce eNOS phosphorylation during Ser1177 and NO production. In vitro, eNOS activity in endothelial cells isolated from PAH lungs was inhibited via the PKC-induced phosphorylation of eNOS-T495, an inhibition site. Plexiform legions in PAH patient lungs confirmed increased pT495-eNOS compared to controls. The pharmacological blockade of PKC activity restored nitric oxide formation in PAH EC [50]. eNOS also couples with caveolin-1 and dissociates from heat shock protein 90 (HSP90) in the hypoxic pulmonary artery, leading to eNOS inactivity [49]. In pulmonary arterial endothelial cells cultured from fetal lambs, mitochondrial uncoupling decreased cellular ATP and reduced both eNOS–HSP90 interactions and nitric oxide signaling [45]. Also, eNOS activity in endothelial cells in PAH lungs was inhibited due to T495 phosphorylation via protein kinase C (PKC). Several in vitro studies identified that PKC phosphorylates T495 and hinders eNOS activity and nitric oxide release [51-53]. Thus, eNOS localization and coupling, as well as eNOS T495 phosphorylation, may regulate decreased nitric oxide production in PAH HPAECs, as opposed to increased eNOS O-GlcNAcylation.

This study shows similarities to our previous study on endothelial cells exposed to steady laminar and oscillating disturbed flow [43]. In both studies, endothelial cells in the diseased state (PAH, disturbed flow) showed increased glucose uptake as well as higher glycolytic metabolite abundance. Both PAH HPAECs and endothelial cells in disturbed flow have increased proliferation. Since endothelial cell proliferation is driven by glycolysis [54,55], increased metabolic activity in both types of diseased cells may relate to increased proliferation. These two cases are also similar in that eNOS O-GlcNAcylation was not regulated by OGT or OGA in endothelial cells, unlike studies in other cell types [20,38,56,57]. Alternative processes such as overall glycolytic flux or GFAT activity [58] may regulate eNOS O-GlcNAcylation instead.

There are also key differences between this study and our prior work [43]. In PAH HPAECs, elevated glycolytic activity did not increase UDP-GlcNAc as it did in human umbilical vein endothelial cells exposed to oscillating disturbed flow. Thus, in PAH, glucose metabolism may progress down different glycolytic side branch pathways. In diabetes, another disease state of increased glucose metabolism, 30% of glucose goes through the polyol pathway [46,59]. The polyol pathway reduces NAPDH [60] while increasing NADH [61]. An increase in the NADH/NAD+ ratio depletes reduced glutathione, an antioxidant, and leads to oxidative stress in diabetic mice [62]. NADH can also increase the formation of the advanced glycation end product (AGE)-forming compound methylglyoxal [63]. Increased ROS and AGE will result in oxidative stress. In chronic hypoxia-induced PAH animal models, pulmonary arteries showed increased ROS production, leading to oxidative stress [64-66]. Therefore, the increased glucose uptake in PAH HPAECs could be metabolized via the polyol pathway and contribute to oxidative stress, or it could be metabolized via the pentose phosphate pathway to counteract oxidative stress.

One of the most important findings in our study is that female HPAECs, especially in PAH donors, showed higher glucose consumption and eNOS O-GlcNAcylation. PAH predominantly affects women [3]; therefore, these differences in female versus male HPAECs could contribute to the sexual dimorphism associated with this disease. In mammals, the single gene encoding OGT is located on the X-chromosome [67], which could explain potential sex differences in O-GlcNAcylation. We and others have shown differences in female and male endothelial cells in culture, specifically in the stress response [68,69]. While these preliminary data highlight interesting sex differences, future studies are needed to examine mechanisms.

While our study shows that PAH HPAECs have elevated glycolytic activity, decreased nitric oxide production, and reduced eNOS O-GlcNAcylation in vitro, this is not without limitations. The biggest limitations are the limited donor number and high donor variability in both non-PAH and PAH HPAECs. This should be considered as a pilot investigation, and more donors should be included in future studies. The average donor age was also different for the non-PAH and PAH donors (44.5 vs. 33.3 years old). We were unable to obtain cells from patients who were any closer in age, and it is therefore possible that the differences we observed could be due to the age difference between the non-PAH and PAH cells. Additionally, these studies were conducted in vitro using HPAECs in static culture on tissue culture polystyrene plates. In the future, pulmonary endothelial cells could be examined in physiological flow conditions, on substrates of different stiffness, and in animal PAH models or intact pulmonary arteries.

## Figures and Tables

**Table 1.** HPAEC donor demographics.

	Disease	Gender	Age	Race
H1	Donor	Female	36	White
H2	Donor	Female	50	White
H3	Donor	Female	34	White
H4	Donor	Male	47	White
H5	Donor	Male	51	White
H6	Donor	Male	49	White
P1	IPAH	Male	40	White
P2	IPAH	Male	51	White
P3	IPAH	Male	16	White
P4	IPAH	Female	32	White
P5	IPAH	Female	16	White
P6	IPAH	Female	45	Unknown

## Data Availability

The data presented in this study are available on request from the corresponding author.

## References

[1] Montani D; Günther S; Dorfmüller P; Perros F; Girerd B; Garcia G; Jaïs X; Savale L; Artaud-Macari E; Price LC; Pulmonary arterial hypertension. Orphanet J. Rare Dis 2013, 8, 97. 23829793 10.1186/1750-1172-8-97 PMC3750932

[2] Hoeper MMP; Humbert MP; Souza RP; Idrees MP; Kawut SMP; Sliwa-Hahnle KP; Jing Z-CP; Gibbs JSRP A global view of pulmonary hypertension. Lancet Respir. Med 2016, 4, 306–322. 26975810 10.1016/S2213-2600(15)00543-3

[3] Pugh ME; Hemnes AR Pulmonary hypertension in women. Expert Rev. Cardiovasc. Ther 2010, 8, 1549–1558. 21090930 10.1586/erc.10.137 PMC3077035

[4] Sakao S; Tatsumi K; Voelkel NF Endothelial cells and pulmonary arterial hypertension: Apoptosis, proliferation, interaction and transdifferentiation. Respir. Res 2009, 10, 95. 19825167 10.1186/1465-9921-10-95 PMC2768704

[5] Cool CD; Stewart JS; Werahera P; Miller GJ; Williams RL; Voelkel NF; Tuder RM Three-dimensional reconstruction of pulmonary arteries in plexiform pulmonary hypertension using cell-specific markers. Evidence for a dynamic and heterogeneous process of pulmonary endothelial cell growth. Am. J. Pathol 1999, 155, 411–419. 10433934 10.1016/S0002-9440(10)65137-1 PMC1866857

[6] Klinger JR; Abman SH; Gladwin MT Nitric Oxide Deficiency and Endothelial Dysfunction in Pulmonary Arterial Hypertension. Am. J. Respir. Crit. Care Med 2013, 188, 639–646. 23822809 10.1164/rccm.201304-0686PP

[7] Giaid A; Saleh D Reduced Expression of Endothelial Nitric Oxide Synthase in the Lungs of Patients with Pulmonary Hypertension. N. Engl. J. Med 1995, 333, 214–221. 7540722 10.1056/NEJM199507273330403

[8] Giaid A; Yanagisawa M; Langleben D; Michel RP; Levy R; Shennib H; Kimura S; Masaki T; Duguid WP; Stewart DJ Expression of Endothelin-1 in the Lungs of Patients with Pulmonary Hypertension. N. Engl. J. Med 1993, 328, 1732–1739. 8497283 10.1056/NEJM199306173282402

[9] Botney MD Role of Hemodynamics in Pulmonary Vascular Remodeling. Am. J. Respir. Crit. Care Med 1999, 159, 361–364. 9927344 10.1164/ajrccm.159.2.9805075

[10] Tuder RM; Davis LA; Graham BB Targeting Energetic Metabolism A New Frontier in the Pathogenesis and Treatment of Pulmonary Hypertension. Am. J. Respir. Crit. Care Med 2012, 185, 260–266. 22077069 10.1164/rccm.201108-1536PP PMC3297113

[11] Dromparis P; Sutendra G; Michelakis ED The role of mitochondria in pulmonary vascular remodeling. J. Mol. Med 2010, 88, 1003–1010. 20734021 10.1007/s00109-010-0670-x

[12] Jonigk D; Golpon H; Bockmeyer CL; Maegel L; Hoeper MM; Gottlieb J; Nickel N; Hussein K; Maus U; Lehmann U; Plexiform lesions in pulmonary arterial hypertension composition, architecture, and microenvironment. Am. J. Pathol 2011, 179, 167–179. 21703400 10.1016/j.ajpath.2011.03.040 PMC3123793

[13] Cool CD; Kennedy D; Voelkel NF; Tuder RM Pathogenesis and evolution of plexiform lesions in pulmonary hypertension associated with scleroderma and human immunodeficiency virus infection. Hum. Pathol 1997, 28, 434–442. 9104943 10.1016/s0046-8177(97)90032-0

[14] Lee SD; Shroyer KR; Markham NE; Cool CD; Voelkel NF; Tuder RM Monoclonal endothelial cell proliferation is present in primary but not secondary pulmonary hypertension. J. Clin. Investig 1998, 101, 927–934. 9486960 10.1172/JCI1910 PMC508641

[15] Tuder RM; Chacon M; Alger L; Wang J; Taraseviciene-Stewart L; Kasahara Y; Cool CD; Bishop AE; Geraci M; Semenza GL; Expression of angiogenesis-related molecules in plexiform lesions in severe pulmonary hypertension: Evidence for a process of disordered angiogenesis. J. Pathol 2001, 195, 367–374. 11673836 10.1002/path.953

[16] Girgis RE; Champion HC; Diette GB; Johns RA; Permutt S; Sylvester JT Decreased exhaled nitric oxide in pulmonary arterial hypertension: Response to bosentan therapy. Am. J. Respir. Crit. Care Med 2005, 172, 352–357. 15879413 10.1164/rccm.200412-1684OC

[17] Khoo Jeffrey P; Zhao L; Alp Nicholas J; Bendall Jennifer K; Nicoli T; Rockett K; Wilkins Martin R; Channon Keith M Pivotal Role for Endothelial Tetrahydrobiopterin in Pulmonary Hypertension. Circulation 2005, 111, 2126–2133. 15824200 10.1161/01.CIR.0000162470.26840.89

[18] Bauer PM; Bauer EM; Rogers NM; Yao M; Feijoo-Cuaresma M; Pilewski JM; Champion HC; Zuckerbraun BS; Calzada MJ; Isenberg JS Activated CD47 promotes pulmonary arterial hypertension through targeting caveolin-1. Cardiovasc. Res 2012, 93, 682–693. 22215724 10.1093/cvr/cvr356 PMC3291089

[19] McLaughlin VV; Archer SL; Badesch DB; Barst RJ; Farber HW; Lindner JR; Mathier MA; McGoon MD; Park MH; Rosenson RS; ACCF/AHA 2009 expert consensus document on pulmonary hypertension a report of the American College of Cardiology Foundation Task Force on Expert Consensus Documents and the American Heart Association developed in collaboration with the American College of Chest Physicians; American Thoracic Society, Inc.; and the Pulmonary Hypertension Association. J. Am. Coll. Cardiol 2009, 53, 1573–1619. 19389575 10.1016/j.jacc.2009.01.004

[20] Barnes JW; Tian L; Heresi GA; Farver CF; Asosingh K; Comhair SA; Aulak KS; Dweik RA O-linked beta-N-acetylglucosamine transferase directs cell proliferation in idiopathic pulmonary arterial hypertension. Circulation 2015, 131, 1260–1268. 25663381 10.1161/CIRCULATIONAHA.114.013878 PMC4390469

[21] Fijalkowska I; Xu W; Comhair SAA; Janocha AJ; Mavrakis LA; Krishnamachary B; Zhen L; Mao T; Richter A; Erzurum SC; Hypoxia inducible-factor1alpha regulates the metabolic shift of pulmonary hypertensive endothelial cells. Am. J. Pathol 2010, 176, 1130. 20110409 10.2353/ajpath.2010.090832 PMC2832136

[22] Xu WL; Kaneko FT; Zheng S; Comhair SAA; Janocha AJ; Goggans T; Thunnissen F; Farver C; Hazen SL; Jennings C; Increased arginase II and decreased NO synthesis in endothelial cells of patients with pulmonary arterial hypertension. FASEB J. 2004, 18, 1746. 15364894 10.1096/fj.04-2317fje

[23] Chen Z; Liu M; Li L; Chen L Involvement of the Warburg effect in non-tumor diseases processes. J. Cell. Physiol 2018, 233, 2839–2849. 28488732 10.1002/jcp.25998

[24] Gurtu V; Michelakis ED Emerging therapies and future directions in pulmonary arterial hypertension. Can. J. Cardiol 2015, 31, 489–501. 25840098 10.1016/j.cjca.2015.01.028

[25] Rai PR; Cool CD; King JA; Stevens T; Burns N; Winn RA; Kasper M; Voelkel NF The cancer paradigm of severe pulmonary arterial hypertension. Am. J. Respir. Crit. Care Med 2008, 178, 558–564. 18556624 10.1164/rccm.200709-1369PP PMC2542431

[26] Wu D; Archer SL Pulmonary hypertension begets pulmonary hypertension: Mutually reinforcing roles for haemodynamics, inflammation, and cancer-like phenotypes. Cardiovasc. Res 2016, 111, 1–4. 27216865 10.1093/cvr/cvw110 PMC4909166

[27] Marsboom G; Wietholt C; Haney CR; Toth PT; Ryan JJ; Morrow E; Thenappan T; Bache-Wiig P; Piao L; Paul J; Lung (1)(8) F-fluorodeoxyglucose positron emission tomography for diagnosis and monitoring of pulmonary arterial hypertension. Am. J. Respir. Crit. Care Med 2012, 185, 670–679. 22246173 10.1164/rccm.201108-1562OC PMC3326289

[28] Bond MR; Hanover JA A little sugar goes a long way: The cell biology of O-GlcNAc. J. Cell Biol 2015, 208, 869–880. 25825515 10.1083/jcb.201501101 PMC4384737

[29] Karunakaran U; Jeoung NH O-GlcNAc Modification: Friend or Foe in Diabetic Cardiovascular Disease. Korean Diabetes J. 2010, 34, 211–219. 20835337 10.4093/kdj.2010.34.4.211 PMC2932889

[30] Mäkimattila S; Virkamäki A; Groop PH; Cockcroft J; Utriainen T; Fagerudd J; Yki-Järvinen H Chronic hyperglycemia impairs endothelial function and insulin sensitivity via different mechanisms in insulin-dependent diabetes mellitus. Circulation 1996, 94, 1276–1282. 8822980 10.1161/01.cir.94.6.1276

[31] Medford HM; Chatham JC; Marsh SA Chronic ingestion of a Western diet increases O-linked-beta-N-acetylglucosamine (O-GlcNAc) protein modification in the rat heart. Life Sci. 2012, 90, 883–888. 22575823 10.1016/j.lfs.2012.04.030 PMC3372663

[32] Musicki B; Kramer MF; Becker RE; Burnett AL Inactivation of phosphorylated endothelial nitric oxide synthase (Ser-1177) by O-GIcNAc in diabetes-associated erectile dysfunction. Proc. Natl. Acad. Sci. USA 2005, 102, 11870–11875. 16085713 10.1073/pnas.0502488102 PMC1187969

[33] Torres C-R; Hart G-W opography and polypeptide distribution of terminal N-acetylglucosamine residues on the surfaces of intact lymphocytes. Evidence for O-linked GlcNAc. J. Biol. Chem 1984, 259, 3308–3317. 6421821

[34] Yki-Järvinen H; Vogt C; Iozzo P; Pipek R; Daniels MC; Virkamäki A; Mäkimattila S; Mandarino L; DeFronzo RA; McClain D; UDP-N-acetylglucosamine transferase and glutamine: Fructose 6-phosphate amidotransferase activities in insulin-sensitive tissues. Diabetologia 1997, 40, 76–81. 9028721 10.1007/s001250050645

[35] Marsh SA; Dell’Italia LJ; Chatham JC Activation of the hexosamine biosynthesis pathway and protein O-GlcNAcylation modulate hypertrophic and cell signaling pathways in cardiomyocytes from diabetic mice. Amino Acids 2011, 40, 819–828. 20676904 10.1007/s00726-010-0699-8 PMC3025273

[36] Federici M; Menghini R; Mauriello A; Hribal ML; Ferrelli F; Lauro D; Sbraccia P; Spagnoli LG; Sesti G; Lauro R Insulin-dependent activation of endothelial nitric oxide synthase is impaired by O-linked glycosylation modification of signaling proteins in human coronary endothelial cells. Circulation 2002, 106, 466–472. 12135947 10.1161/01.cir.0000023043.02648.51

[37] Clark RJ; McDonough PM; Swanson E; Trost SU; Suzuki M; Fukuda M; Dillmann WH Diabetes and the accompanying hyperglycemia impairs cardiomyocyte calcium cycling through increased nuclear O-GlcNAcylation. J. Biol. Chem 2003, 278, 44230–44237. 12941958 10.1074/jbc.M303810200

[38] Hu Y; Belke D; Suarez J; Swanson E; Clark R; Hoshijima M; Dillmann Wolfgang H Adenovirus-Mediated Overexpression of O-GlcNAcase Improves Contractile Function in the Diabetic Heart. Circ. Res 2005, 96, 1006–1013. 15817886 10.1161/01.RES.0000165478.06813.58

[39] Luo B; Soesanto Y; McClain DA Protein modification by O-linked GlcNAc reduces angiogenesis by inhibiting Akt activity in endothelial cells. Arter. Thromb. Vasc. Biol 2008, 28, 651–657. 10.1161/ATVBAHA.107.159533 PMC2734484 18174452

[40] Du X; Matsumura T; Edelstein D; Rossetti L; Zsengeller Z; Szabo C; Brownlee M Inhibition of GAPDH activity by poly(ADP-ribose) polymerase activates three major pathways of hyperglycemic damage in endothelial cells. J. Clin. Investig 2003, 112, 1049–1057. 14523042 10.1172/JCI18127 PMC198524

[41] Yao D; Taguchi T; Matsumura T; Pestell R; Edelstein D; Giardino I; Suske G; Rabbani N; Thornalley PJ; Sarthy VP; High glucose increases angiopoietin-2 transcription in microvascular endothelial cells through methylglyoxal modification of mSin3A. J. Biol. Chem 2007, 282, 31038–31045. 17670746 10.1074/jbc.M704703200

[42] Du XL; Edelstein D; Dimmeler S; Ju QD; Sui C; Brownlee M Hyperglycemia inhibits endothelial nitric oxide synthase activity by posttranslational modification at the Akt site. J. Clin. Investig 2001, 108, 1341–1348. 11696579 10.1172/JCI11235 PMC209429

[43] Basehore SE; Bohlman S; Weber C; Swaminathan S; Zhang Y; Jang C; Arany Z; Clyne AM Laminar Flow on Endothelial Cells Suppresses eNOS O-GlcNAcylation to Promote eNOS Activity. Circ. Res 2021, 129, 1054–1066. 34605247 10.1161/CIRCRESAHA.121.318982 PMC8653916

[44] Jang C; Hui S; Lu W; Cowan AJ; Morscher RJ; Lee G; Liu W; Tesz GJ; Birnbaum MJ; Rabinowitz JD The Small Intestine Converts Dietary Fructose into Glucose and Organic Acids. Cell Metab. 2018, 27, 351–361.e3. 29414685 10.1016/j.cmet.2017.12.016 PMC6032988

[45] Sharma S; Sud N; Wiseman DA; Carter AL; Kumar S; Hou Y; Rau T; Wilham J; Harmon C; Oishi P; Altered carnitine homeostasis is associated with decreased mitochondrial function and altered nitric oxide signaling in lambs with pulmonary hypertension. Am. J. Physiol. Lung Cell. Mol. Physiol 2008, 294, L46–L56. 18024721 10.1152/ajplung.00247.2007 PMC3970936

[46] Clyne AM Endothelial response to glucose: Dysfunction, metabolism, and transport. Biochem. Soc. Trans 2021, 49, 313–325. 33522573 10.1042/BST20200611 PMC7920920

[47] Moiz B; Garcia J; Basehore S; Sun A; Li A; Padmanabhan S; Albus K; Jang C; Sriram G; Clyne AM (13)C Metabolic Flux Analysis Indicates Endothelial Cells Attenuate Metabolic Perturbations by Modulating TCA Activity. Metabolites 2021, 11, 226. 33917224 10.3390/metabo11040226 PMC8068087

[48] Sedlak JM; Clyne AM A Modified Parallel Plate Flow Chamber to Study Local Endothelial Response to Recirculating Disturbed Flow. J. Biomech. Eng 2020, 142, 0410031. 31536122 10.1115/1.4044899 PMC7104763

[49] Murata T; Sato K; Hori M; Ozaki H; Karaki H Decreased endothelial nitric-oxide synthase (eNOS) activity resulting from abnormal interaction between eNOS and its regulatory proteins in hypoxia-induced pulmonary hypertension. J. Biol. Chem 2002, 277, 44085–44092. 12185080 10.1074/jbc.M205934200

[50] Ghosh S; Gupta M; Xu W; Mavrakis DA; Janocha AJ; Comhair SAA; Haque MM; Stuehr DJ; Yu J; Polgar P; Phosphorylation inactivation of endothelial nitric oxide synthesis in pulmonary arterial hypertension. Am. J. Physiol. Lung Cell. Mol. Physiol 2016, 310, L1199–L1205. 27130529 10.1152/ajplung.00092.2016 PMC4935470

[51] Fleming I; Fisslthaler B; Dimmeler S; Kemp BE; Busse R Phosphorylation of Thr495 regulates Ca^2+^/calmodulin-dependent endothelial nitric oxide synthase activity. Circ. Res 2001, 88, e68–e75. 11397791 10.1161/hh1101.092677

[52] Fleming I; Busse R Signal transduction of eNOS activation. Cardiovasc. Res 1999, 43, 532–541. 10690325 10.1016/s0008-6363(99)00094-2

[53] Matsubara M; Hayashi N; Jing T; Titani K Regulation of endothelial nitric oxide synthase by protein kinase C. J. Biochem 2003, 133, 773–781. 12869534 10.1093/jb/mvg099

[54] De Bock K; Georgiadou M; Schoors S; Kuchnio A; Wong BW; Cantelmo AR; Quaegebeur A; Ghesquiere B; Cauwenberghs S; Eelen G; Role of PFKFB3-driven glycolysis in vessel sprouting. Cell 2013, 154, 651–663. 23911327 10.1016/j.cell.2013.06.037

[55] Schoors S; De Bock K; Cantelmo AR; Georgiadou M; Ghesquiere B; Cauwenberghs S; Kuchnio A; Wong BW; Quaegebeur A; Goveia J; Partial and transient reduction of glycolysis by PFKFB3 blockade reduces pathological angiogenesis. Cell Metab. 2014, 19, 37–48. 24332967 10.1016/j.cmet.2013.11.008

[56] Watson LJ; Facundo HT; Ngoh GA; Ameen M; Brainard RE; Lemma KM; Long BW; Prabhu SD; Xuan Y-T; Jones SP O-linked β-N-acetylglucosamine transferase is indispensable in the failing heart. Proc. Natl. Acad. Sci. USA 2010, 107, 17797–17802. 20876116 10.1073/pnas.1001907107 PMC2955091

[57] Belke DD Swim-exercised mice show a decreased level of protein O-GlcNAcylation and expression of O-GlcNAc transferase in heart. J. Appl. Physiol 2011, 111, 157–162. 21493720 10.1152/japplphysiol.00147.2011

[58] da Costa RM; da Silva JF; Alves JV; Dias TB; Rassi DM; Garcia LV; Lobato N.d.S.; Tostes RC Increased O-GlcNAcylation of Endothelial Nitric Oxide Synthase Compromises the Anti-contractile Properties of Perivascular Adipose Tissue in Metabolic Syndrome. Front. Physiol 2018, 9, 341. 29681862 10.3389/fphys.2018.00341 PMC5897513

[59] Giacco F; Brownlee M Oxidative stress and diabetic complications. Circ. Res 2010, 107, 1058–1070. 21030723 10.1161/CIRCRESAHA.110.223545 PMC2996922

[60] Yan L-J Redox imbalance stress in diabetes mellitus: Role of the polyol pathway. Anim. Models Exp. Med 2018, 1, 7–13. 10.1002/ame2.12001 PMC5975374 29863179

[61] Williamson JR; Chang K; Frangos M; Hasan KS; Ido Y; Kawamura T; Nyengaard JR; van den Enden M; Kilo C; Tilton RG Hyperglycemic pseudohypoxia and diabetic complications. Diabetes 1993, 42, 801–813. 8495803 10.2337/diab.42.6.801

[62] Chung SSM; Ho ECM; Lam KSL; Chung SK Contribution of Polyol Pathway to Diabetes-Induced Oxidative Stress. J. Am. Soc. Nephrol 2003, 14, S233. 12874437 10.1097/01.asn.0000077408.15865.06

[63] Pugliese G; Tilton RG; Williamson JR Glucose-induced metabolic imbalances in the pathogenesis of diabetic vascular disease. Diabetes/Metab. Rev 1991, 7, 35–59. 1935535 10.1002/dmr.5610070106

[64] Fresquet F; Pourageaud F; Leblais V; Brandes RP; Savineau JP; Marthan R; Muller B Role of reactive oxygen species and gp91phox in endothelial dysfunction of pulmonary arteries induced by chronic hypoxia. Br. J. Pharmacol 2006, 148, 714–723. 16715116 10.1038/sj.bjp.0706779 PMC1751862

[65] Hoshikawa Y; Ono S; Suzuki S; Tanita T; Chida M; Song C; Noda M; Tabata T; Voelkel NF; Fujimura S Generation of oxidative stress contributes to the development of pulmonary hypertension induced by hypoxia. J. Appl. Physiol 2001, 90, 1299–1306. 11247927 10.1152/jappl.2001.90.4.1299

[66] Liu JQ; Zelko IN; Erbynn EM; Sham JS; Folz RJ Hypoxic pulmonary hypertension: Role of superoxide and NADPH oxidase (gp91phox). Am. J. Physiol. Lung Cell. Mol. Physiol 2006, 290, L2–L10. 16085672 10.1152/ajplung.00135.2005

[67] Olivier-Van Stichelen S; Abramowitz LK; Hanover JA X marks the spot: Does it matter that O-GlcNAc transferase is an X-linked gene? Biochem. Biophys. Res. Commun 2014, 453, 201–207. 24960196 10.1016/j.bbrc.2014.06.068 PMC4253714

[68] Weber CM; Clyne AM Sex differences in the blood-brain barrier and neurodegenerative diseases. APL Bioeng. 2021, 5, 011509. 33758788 10.1063/5.0035610 PMC7968933

[69] Weber CM; Harris MN; Zic SM; Sangha GS; Arnold NS; Dluzen DF; Clyne AM Angiotensin II Increases Oxidative Stress and Inflammation in Female, But Not Male, Endothelial Cells. Cell. Mol. Bioeng 2023, 16, 127–141. 37096068 10.1007/s12195-023-00762-2 PMC10121986

